# Some Points of View of the Medical Profession

**Published:** 1912-03-23

**Authors:** 


### THE EDITOR'S LETTER-BOX.

### COMPETITIONS FOR HOSPITAL BUILDINGS: FURTHER OPINIONS.

### SOME POINTS OF VIEW OF THE MEDICAL PROFESSION.

Sir,—I am only a member of the medical profession who has to work in two hospitals, and my views on hospital architecture are those solely of one who uses hospital buildings as part of the treatment of the sick. I know nothing of architecture; but I have to put the architect's puddings to the practical proof of eating them, and I ask leave to comment shortly on the remarkable letter of Mr. A. S. Snell, published in The Hospital of March 16.

This letter seems to me to beg every question raised in your original article, and to evince exactly that spirit of prejudice and exclusiveness which is responsible for so many of the muddles and blunders to be seen in hospital buildings all over England. He says "the pros and cons of a purely architectural question such as this is [*sic*] scarcely likely to be of interest to the majority of your readers." I am sure that in this opinion he is totally wrong; but I am more concerned at the moment to deal with the first half of this sentence. The building of a hospital is, in a sense, a "purely architectural question"; but the architect who designs a hospital is bound to consider the purposes for which his building is wanted and the uses to which it is to be put. He who ignores all such considerations, on the ground that he is a "pure architect," may receive the encomiums of Mr. Snell for his architecture, but surely not those of anyone else, either in the profession of architecture or outside it.

Then Mr. Snell dismisses all the pertinent criticisms of your two correspondents as convincing only to "unsuccessful competitors." This sneer does not strike an outsider like myself as any answer to the detailed and, if unanswered, damaging criticisms put forward by your correspondents (and confirmed, I notice, by another F.R.I.B.A. in your issue of March 16).

Yet again Mr. Snell remarks that an assessor who has given many hours to the study of the plans must be a better judge of them than your anonymous correspondents. It does not follow in the least that this is so; for it depends not only on the competence of the assessor, which we will assume, but also upon an experience of the practical working of a hospital, and this even eminent architects very seldom possess. The whole argument is that an assessor ought to have an expert acquaintance with hospital working and requirements; and that if he has not got it, a joint assessor who has should be appointed. This is an intelligible and a sensible proposition; and if the "pure architects" continue to flout commonsense and hospital requirements in designing hospitals, it is a reform which they will have no one to blame for but themselves.

Finally, may I justify the interest I feel in this matter by remarking on the excellent sense of the architectural articles which frequently appear in your columns. These articles have often made me realise the avoidable shortcomings of the (London) hospitals in which I work, and have impressed on me the very point now in dispute, viz. the need for assessors who know something about hospital administration as well as those who judge the strictly architectural features of new designs for hospital buildings. As my interest in the present dispute is entirely that of an onlooker, I ask to be allowed to remain anonymous.—Yours faithfully,

London, S.W. Hospital Medical Officer.

